# Contrast-Enhanced Magnetic Resonance Angiography Using a Novel Elastin-Specific Molecular Probe in an Experimental Animal Model

**DOI:** 10.1155/2018/9217456

**Published:** 2018-10-23

**Authors:** Carolin Reimann, Julia Brangsch, Jan Ole Kaufmann, Lisa C. Adams, David C. Onthank, Simon P. Robinson, Rene M. Botnar, Federico Collettini, Marcus R. Makowski

**Affiliations:** ^1^Charité–Universitätsmedizin Berlin, Corporate Member of Freie Universität Berlin, Humboldt-Universität zu Berlin, and Berlin Institute of Health, Charitéplatz 1, 10117 Berlin, Germany; ^2^Department of Veterinary Medicine, Institute of Animal Welfare, Animal Behavior and Laboratory Animal Science, Freie Universität Berlin, Königsweg 67, Building 21, 14163 Berlin, Germany; ^3^Lantheus Medical Imaging, North Billerica, MA, USA; ^4^King's College London, School of Biomedical Engineering and Imaging Sciences United Kingdom, St Thomas' Hospital, Westminster Bridge Road, London SE1 7EH, UK; ^5^BHF Centre of Excellence, King's College London, United Kingdom, Denmark Hill Campus, 125 Coldharbour Lane, London SE5 9NU, UK; ^6^Wellcome Trust and EPSRC Medical Engineering Center, King's College London, Gibbs Building, 215 Euston Road, London NW1 2BE, UK; ^7^Escuela de Ingeniería, Pontificia Universidad Católica de Chile, Santiago, Chile

## Abstract

**Objectives:**

The aim of this study was to test the potential of a new elastin-specific molecular agent for the performance of contrast-enhanced first-pass and 3D magnetic resonance angiography (MRA), compared to a clinically used extravascular contrast agent (gadobutrol) and based on clinical MR sequences.

**Materials and Methods:**

Eight C57BL/6J mice (BL6, male, aged 10 weeks) underwent a contrast-enhanced first-pass and 3D MR angiography (MRA) of the aorta and its main branches. All examinations were on a clinical 3 Tesla MR system (Siemens Healthcare, Erlangen, Germany). The clinical dose of 0.1 mmol/kg was administered in both probes. First, a time-resolved MRA (TWIST) was acquired during the first-pass to assess the arrival and washout of the contrast agent bolus. Subsequently, a high-resolution 3D MRA sequence (3D T1 FLASH) was acquired. Signal-to-noise ratios (SNRs) and contrast-to-noise ratios (CNRs) were calculated for all sequences.

**Results:**

The elastin-specific MR probe and the extravascular imaging agent (gadobutrol) enable high-quality MR angiograms in all animals. During the first-pass, the probes demonstrated a comparable peak enhancement (300.6 ± 32.9 vs. 288.5 ± 33.1, *p* > 0.05). Following the bolus phase, both agents showed a comparable intravascular enhancement (SNR: 106.7 ± 11 vs. 102.3 ± 5.3; CNR 64.5 ± 7.4 vs. 61.1 ± 7.2, *p* > 0.05). Both agents resulted in a high image quality with no statistical difference (*p* > 0.05).

**Conclusion:**

The novel elastin-specific molecular probe enables the performance of first-pass and late 3D MR angiography with an intravascular contrast enhancement and image quality comparable to a clinically used extravascular contrast agent.

## 1. Introduction

Contrast-enhanced magnetic resonance angiography (CE-MRA) has become the clinical reference technique for the evaluation of vascular territories and of associated pathologies in patients [1, 2]. CE-MRA angiography is performed, e.g., for the visualization of the aorta, the carotid arteries, the renal arteries, and most other arterial territories [1, 3]. Therefore, different techniques are currently in clinical use, including the first-pass angiography technique, which is usually performed in a single breath-hold. For this technique, the timing of the arterial bolus is of high importance to achieve the maximum arterial enhancement. Additionally, in most imaging protocols different phases are acquired, including the arterial, venous, and portal venous phase, depending on the clinical question [2]. The main advantage of this approach is the imaging of the different vascular phases with a high intravascular enhancement, whereas the main drawback is that due to the short scan time, only relatively large vascular structures can be visualized. A typical clinically used sequence type is the TWIST (time-resolved angiography with interleaved stochastic trajectories) sequence, which improves the temporal resolution of the sequence based on specific stochastic trajectories [4]. In clinical practice, an additional high-resolution 3D angiography (e.g., 3D T1 FLASH) is usually acquired at a relatively late time point with high spatial resolution. This type of sequence enables the visualization of relatively small vascular structures.

Recently, a novel small-molecular-weight gadolinium-based elastin-specific molecular agent has been introduced [5–8]. This probe has been used for different molecular applications, including the characterization of the arterial vascular wall in different vascular diseases, e.g., atherosclerosis, aortic aneurysms, or Marfan's disease [5–8]. Since this agent contains only a small targeting moiety in addition to a single gadolinium complex, this probe is comparable to currently clinically used contrast agents. To take full clinical advantage of the elastin agent, it would be preferable to also be able to gather the early blood phase clinical applications, including magnetic resonance angiographies (MRA).

The aim of this study was to test the potential of a small-molecular-weight gadolinium-based elastin-specific magnetic resonance (MR) probe for the performance of contrast-enhanced first-pass and late 3D MRA using clinical MR protocols in comparison to a clinically used extravascular contrast agent (gadobutrol).

## 2. Materials and Methods

### 2.1. Animal Experiments

All procedures were performed according to the guidelines and regulations of the Federation of Laboratory Animal Science Associations (FELASA) and the local guidelines and provisions for the implementation of the Animal Welfare Act. For imaging, eight ten-week-old male homozygous C57BL/6J mice from Charles River Laboratories (Edinburgh, United Kingdom) were used. All animals were housed in a clean barrier and fed with a standard lab diet. Prior to the imaging sessions, mice were anesthetized using an intraperitoneal administration of a combination of medetomidine (500 *µ*g/kg), fentanyl (50 *µ*g/kg), and midazolam (5 mg/kg). Following the final imaging session, mice were euthanized. For histological examinations, a perfusion at a pressure of 100 mm Hg with the fixative MorFFFix® (Morphisto, Frankfurt am Main, Germany) was performed followed by excision of the carotid arteries, brachiocephalic artery, and aorta including the renal arteries. All animal procedures in this study were conducted by a veterinarian and all possible steps were taken to avoid animal suffering at each stage of the experiment.

### 2.2. Animal Handling and *In Vivo* Magnetic Resonance Imaging

The imaging sessions were performed using a clinical 3T Siemens system (Biograph, Siemens Healthcare, Erlangen, Germany) and a clinical single-loop coil (diameter 4 cm, Siemens Healthcare, Erlangen, Germany). During the imaging sessions, body temperature (37°C) was monitored using a MR-compatible heating system (Model 1025, SA Instruments Inc, Stony Brook, NY). For the administration of the different MR imaging agents, a small diameter tube with an attached needle was inserted into the tail vein of the animals. Following the acquisition of unenhanced scans, the administration of the imaging agents was performed.

### 2.3. Imaging Agents

As a “control” agent, gadobutrol (Gadovist®, Bayer Pharma AG, Berlin, Germany) was administered undiluted at a clinical dose of 0.1 mmol/kg bodyweight and followed by 0.01 ml saline. In a previous study, the *R*1 relaxivity of gadobutrol at 3T was reported to be 3.6 ± 0.2 [9]. The elastin-specific molecular agent (ESMA, Lantheus Medical Imaging, North Billerica, Massachusetts, USA) was administered diluted to the same volume and at the same dose of 0.1 mmol/kg bodyweight. The unbound longitudinal relaxivity of the probe at 3T was reported to be 4.7 ± 0.1 [5]. The imaging agents were administered in a random order in the same animal. To allow for a complete clearance of the imaging agents, a seven-day time gap was left between both imaging sessions. Prior to the second imaging agent administration, an unenhanced scan (prescan) was performed in all imaging sessions in all animals to confirm that no residual agent remained in the vascular system.

### 2.4. Time-Resolved MR Angiography (TWIST) and High-Resolution 3D Angiography (FLASH)

All imaging sequences used in this study were derived from a clinically used MR protocol and adapted for small animal imaging. For image acquisition the following sequence parameters were used. First, a low-resolution localizer scan was performed in sagittal, coronal, and transverse orientation to plan the subsequent imaging planes. The localizer scan had the following parameters: field of view 280 mm, matrix 256, and slice thickness 6 mm. Subsequently, a TWIST (time-resolved angiography with interleaved stochastic trajectories) T1 angiography sequence was performed: Repetition time (TR) 3.5 ms, echo time (TE) 1.3 ms 10 slices, flip angle 12°, 50 timepoints, field of view 316, matrix 480, interpolated inplane resolution 0.3 mm, and slice thickness 0.5 mm. For the late-phase high-resolution angiography, a 3D T1 FLASH (fast low angle shot) sequence was used: TR 4.7 ms, TE 1.9 ms, 60 slices, field of view 316, matrix 480, interpolated inplane resolution 0.3 mm, and slice thickness 0.5 mm.

### 2.5. Image Analysis of MR Angiograms

MR images were analyzed using OsiriX (version 7.1, OsiriX foundation). All images were analyzed in a random order and blinded to the imaging agent and the different time points. Signal-to-noise (SNR) and contrast-to-noise (CNR) measurements were performed in data sets of the time-resolved MR angiography (TWIST) and high-resolution 3D FLASH imaging. To quantify the vascular signal enhancement in the time-resolved TWIST sequence, signal measurements were performed using a region of interest in the left ventricle of the heart. To quantify the vascular signal enhancement in the T1 FLASH sequence, signal measurements were performed using a region of interest in the aortic lumen at the level of the heart. Noise measurements were performed anterior to the thorax with the standard deviation of the region of interest. The SNR was calculated as follows: SNR = mean signal (aortic lumen)/standard deviation signal (noise). For the assessment of the contrast-to-noise ratio, an additional region of interest was placed in the muscle tissue dorsal of the aorta. The CNR was calculated as follows: CNR = (mean signal (aortic lumen) − mean signal (muscle tissue))/standard deviation signal (noise).

For the image quality assessment of the 3D T1 FLASH sequence, the image quality was ranked based on a five-point scale: 1 = poor quality information, 2 = aorta visible but markedly blood, 3 = aorta visible with moderate blurring, 4 = aorta visible with minimal blurring, and 5 = aorta visible with sharply defined borders. The image grading system used was modified based on the system by McConnell et al. [10].

### 2.6. Histological Analysis of Arterial Vessel System

The histological analysis was performed for the aorta, the brachiocephalic artery, and the carotid artery. The vessels were embedded in paraffin and cut into 5 -*µ*m thick serial sections. These sections were subsequently stained with Miller's Elastica van Gieson stain (EvG) and hematoxylin and eosin (HE).

### 2.7. Morphometry of the Arterial System

The complete arterial system including the carotid arteries, the subclavian arteries, brachiocephalic artery, aortic arch, and descending aorta to the iliac arteries was visualized in all MR scans. The vessel bifurcations, e.g., aorta to brachiocephalic artery, brachiocephalic artery to subclavian artery, were used as anatomical landmarks for coregistration of the *in viv*o MR images and *ex vivo* images from histology. The morphometrical analysis was performed using elastin-stained sections (Miller's Elastica van Gieson stain) and ImageJ software (Version 1.51f, ImageJ).

### 2.8. Statistical Analysis

Data are expressed as mean ± standard deviation. To determine continuous variables Student's *t*-test (unpaired, 2-tailed) was used. Linear regression analysis and Bland-Altman analysis were used to compare the two contrast agents. For the first method, the slope of the regression line (b) together with the Pearson correlation coefficient (*R*) was calculated. For the latter method instead, the mean difference (*M*) with limits of agreement (*I*) (±1.96 times the standard deviation) was calculated. *p* < 0.05 was used for significance.

## 3. Results

No side effects or adverse reactions to the imaging agents were observed in the investigated animals.

### 3.1. Time-Resolved First-Pass MR Angiography (TWIST)

**Figure 1:**
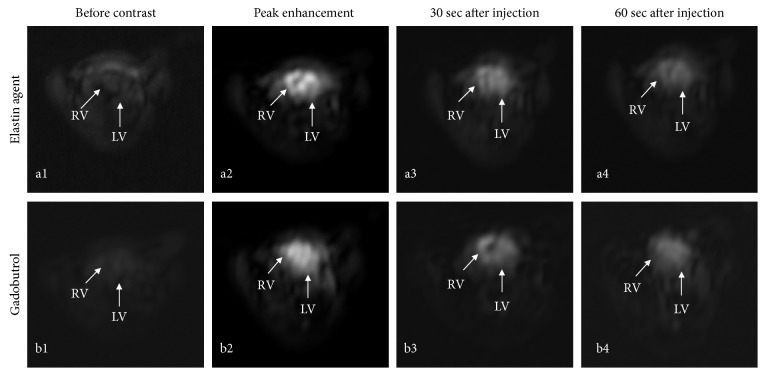
Example of a first-pass MR angiography (TWIST) following the administration of the elastin-specific molecular agent and the clinically used gadobutrol. (a, b) The images show the first-pass MR angiography (TWIST) following the administration of the elastin agent and the clinically used agent gadobutrol. (a1, b1) Precontrast images at the level of the heart show no relevant signal enhancement prior to the administration of the probes. (a2, b2) Immediately after the administration of the imaging agents, a high signal in the left and right ventricle can be appreciated during the first-pass. Visually, the signal intensity from both probes was comparable. (a3, b3) 30 seconds after the administration of the probes, a clear reduction of the signal at the level of the heart can be appreciated. (a4, b4) 60 seconds following the administration of the probes, a further decrease in signal can be appreciated. RV: right ventricle; LV: left ventricle.

**Figure 2:**
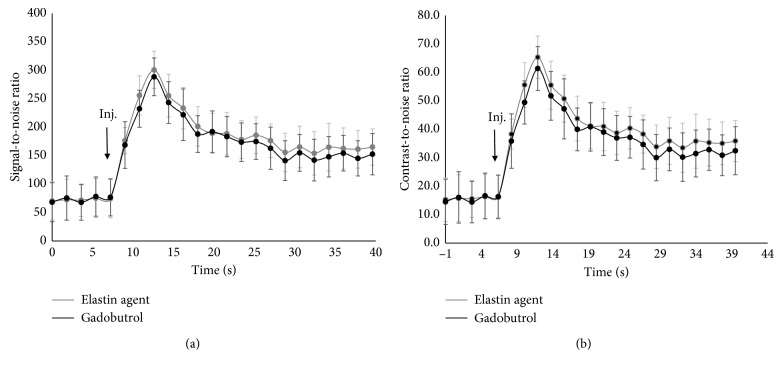
Signal-to-noise ratios and contrast-to-noise ratios in the first-pass MR angiography (TWIST) following the administration of the elastin-specific molecular agent and the clinically used agent gadobutrol. (a, b) Signal-to-noise and contrast-to-noise ratios of the first-pass and the early phases following the administration of the elastin-specific molecular agent and the clinically used gadobutrol (*n*=8). A contrast enhancement curve, typical for extravascular imaging agents, was visualized. Signal-to-noise in contrast-to-noise measurements were performed in the left ventricle. Directly after administration, the peak enhancement was measured during the first-pass. The signal-to-noise in contrast-to-noise ratio derived from the elastin-specific agent was slightly, however not significantly (*p* > 0.05), higher compared to the enhancement derived from gadobutrol. This can be explained by the slightly higher relaxivity of the elastin-specific agent compared to gadobutrol. Following the first-pass, both agents show a comparable biokinetic of the reduction in signal. Inj.: injection of the respective probe.

An example of the dynamic contrast enhancement using the elastin-specific molecular agent and gadobutrol is shown in Figures 1 and 2. The different signal-to-noise ratios (SNRs) and contrast-to-noise ratios (CNRs) derived from the time-resolved MR angiography (TWIST) for the evaluated probes are shown in Figures 2 and 2. A contrast enhancement curve, which is typical for extravascular contrast agents, was visualized. The elastin-specific molecular agent showed a slightly higher peak enhancement at approximately three seconds following the contrast injection, compared to the enhancement of gadobutrol (SNR: 300.6 ± 32.9 vs 288.5 ± 33.1, *p* > 0.05; CNR: 65.3 ± 7.5 vs. 61.4 ± 7.7, *p* > 0.05). For the peak enhancement, the average aortic SNR and CNR was not significantly different between the elastin-specific agent and the extracellular extravascular agent gadobutrol (*p* > 0.05). Also, in the later phases after more than 20 seconds, the average SNR and CNR between the contrast agents was not significantly different from each other (*p* > 0.05) for each time point measured.

### 3.2. High-Resolution 3D Angiography (T1 FLASH)

**Figure 3:**
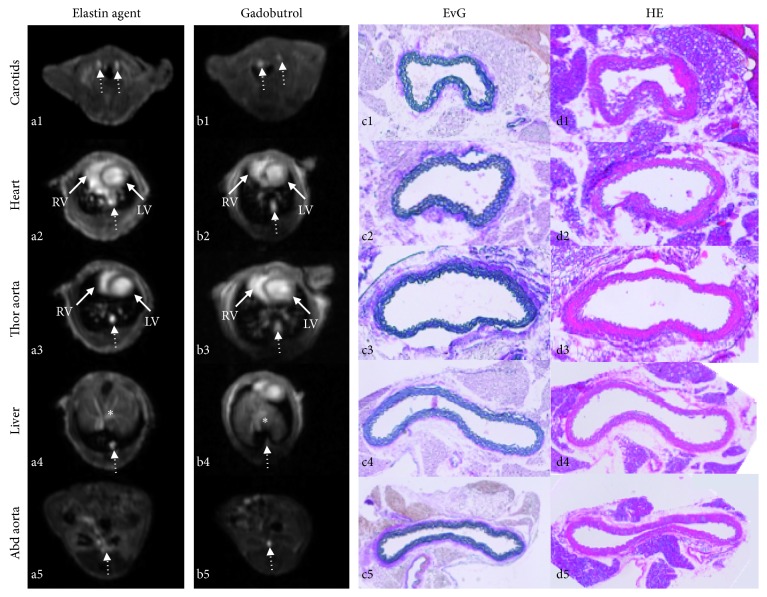
Example of a high-resolution 3D angiography (T1 FLASH) following the administration of the elastin-specific molecular agent and the clinically used agent gadobutrol. (a, b) Transversal imaging planes at different anatomical locations demonstrating the visualization of the large vascular structures. (c, d) Corresponding histology at the different locations. (a1, b2) Visualization of the carotid vasculature with the elastin-specific agent and gadobutrol. It can be appreciated that the carotid arteries can be visualized with a comparable enhancement using both probes. (a2, a3 b2, b3) Visualization of the left/right ventricle and thoracic aorta using both probes. (a4, b4) Visualization of the aorta (dotted area) and the hepatic vasculature (∗). (a5, b5) Visualization of the abdominal aorta at the level of the kidneys. (c1–c5, d1–d5) Corresponding Elastica van Giesson stain and hematoxylin/eosin stain of the aorta at corresponding levels. EvG: Elastica van Giesson stain; HE: hematoxylin/eosin stain; RV: right ventricle; LV: left ventricle.

**Figure 4:**
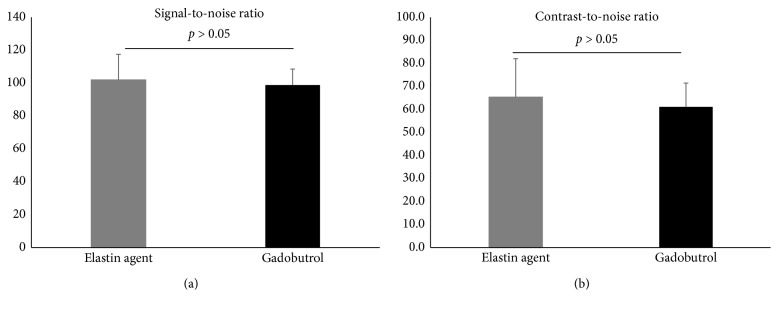
Signal-to-noise ratios and contrast-to-noise ratios in the high-resolution 3D angiography (FLASH) following the administration of the elastin-specific molecular agent and the clinically used agent gadobutrol. (a, b) Signal-to-noise and contrast-to-noise ratios of the aorta in the high-resolution 3D angiography (FLASH) following the administration of the elastin-specific molecular agent and the clinically used gadobutrol (*n*=8). The elastin-specific molecular probe demonstrated a slightly higher signal-to-noise ratio and contrast-to-noise ratio, which was not significantly different from the signal-to-noise ratio and contrast-to-noise ratio of gadobutrol (*p* > 0.05).

A typical high-resolution 3D T1 FLASH angiography is shown in Figures 3 and 3. The SNRs and CNRs in the later bolus phase, approximately three minutes following the injection of the probes, demonstrated slightly higher values for the elastin-specific agent compared to the extracellular extravascular agent gadobutrol (Figure 4). The average SNR and CNR for the elastin-specific agent were 109.2 ± 16.6 and 65.4 ± 8.4. The average SNR and CNR for the extracellular extravascular agent gadobutrol were 105.9 ± 10.4 and 61.1 ± 7.2. No significant differences were measured between the two probes for the SNR (*p* > 0.05) and the CNR (*p* > 0.05).

### 3.3. Image Quality for the High-Resolution 3D FLASH Angiography

**Figure 5:**
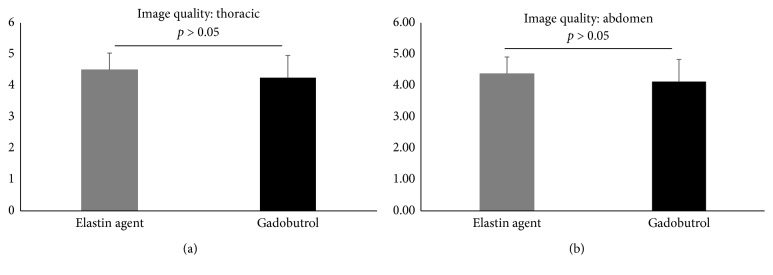
Image quality of the high-resolution 3D angiography (FLASH) following the administration of the elastin-specific molecular agent and the clinically used agent gadobutrol. (a, b) Image quality of the vascular system in the high-resolution 3D angiography (FLASH) following the administration of the elastin-specific molecular agent and the clinically used agent gadobutrol (*n*=8). Both probes showed a high image quality for the visualization of the aorta in the thorax and abdomen with no significant difference between the two probes (*p* > 0.05).

The image quality for both the elastin-specific molecular agent and the extracellular extravascular agent gadobutrol were high for the visualization of the thoracic and the abdominal aorta (Figure 5). For the elastin-specific agent and average image quality of 4.50 ± 0.53 and 4.38 ± 0.52 was measured for the aorta in the thorax and the abdomen. For the extravascular agent gadobutrol, an average image quality of 4.25 ± 0.71 and 4.13 ± 0.64 was measured for the aorta in the thorax and the abdomen. No significant difference was found between the two different imaging agents (*p* ≤ 0.05).

### 3.4. Correlation of *In Vivo* Angiography with *Ex Vivo* Histology

**Figure 6:**
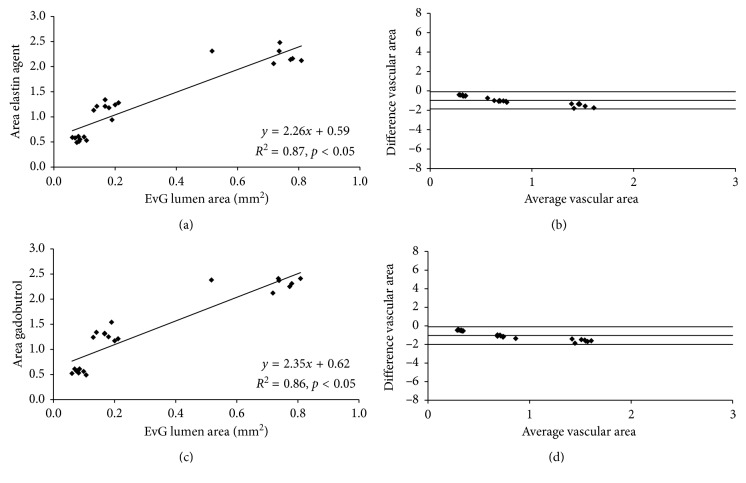
Correlation of *in vivo* aortic area measurements following the administration of the probes and the *ex vivo* measurements on the Elastica van Giesson stain. (a) *In vivo* area measurements following the administration of the elastin-specific molecular probe showed a close correlation with the *ex vivo* measurements on the Elastica van Giesson stain (*n*=4). Measurements were performed on the high-resolution 3D angiography (flash). (b) Bland-Altman analysis only shows a minor difference between the two measurements with relatively small confidence intervals. (c) *In vivo* area measurements following the administration of gadobutrol showed a close correlation with the *ex vivo* measurements on the Elastica van Giesson stain (*n*=4). (d) Bland-Altman analysis only shows a minor difference between the two measurements with relatively small confidence intervals. EvG: Elastica van Giesson stain.

*In vivo* and *ex vivo* luminal area measurements were performed in the high-resolution 3D angiography (T1 FLASH) following the administration of the elastin-specific molecular agent and the extracellular extravascular agent gadobutrol (Figures 3 and 3). *Ex vivo* area measurements were performed based on the Elastica van Giesson stain (Figure 3). A close and significant correlation was measured between the *in vivo* luminal area following the administration of the elastin-specific molecular agent (*y* = 2.26*x* + 0.59, *R* ^2^ = 0.87, *p* < 0.05, Figures 6 and 6). Furthermore, a close and significant correlation was measured between the *in vivo* luminal area following the administration of gadobutrol (*y* = 2.35*x* + 0.62, *R* ^2^ = 0.86, *p* < 0.05, Figures 6 and 6). Small 95% confidence intervals can be seen in the Bland-Altman plots. For the elastin-specific agent, the 95% confidence interval ranged from −1.86 to −0.09. For gadobutrol, the 95% confidence interval ranged from −2.00 to −0.09. Overall, measurements of the *in vivo* vascular area were higher compared to measurements of the *ex vivo* vascular area. These smaller size measurements on *ex vivo* histology can be explained by an *in vivo* partial volume effect and tissue shrinkage, which results from the processing of the tissue samples.

## 4. Discussion

This study demonstrated that a novel elastin-specific molecular probe enables the performance of a contrast-enhanced first-pass and late 3D MR angiography at a clinically relevant dose with an intravascular contrast enhancement and image quality comparable to a clinically used extravascular contrast agent (gadobutrol). All measurements were performed at the same clinical dose of both probes and at a clinical field strength with a clinical sequence design.

### 4.1. Biokinetic Properties of the Elastin-Specific Probe

The paramagnetic ^158^Gd-labelled C_32_H_40_N_7_O_11_ (856 Dalton) elastin-specific molecular probe is a low-molecular-weight contrast agent. Comparable to clinically used gadolinium-based contrast agents, the relatively small probe is labelled with a single gadolinium chelate [5]. Because of its small size, this probe shows a rapid clearance from the blood pool, comparable to clinically used first-pass agents. To enable elastin imaging of the molecular target shortly after the intravenous administration, a high target to background ratio was shown to be reached within the first hour [5]. For the elastin-specific agent, the uptake in the target tissue was demonstrated to be the highest at approximately 30 to 45 minutes after injection. Nontarget tissues, such as heart, lung and muscle, showed a low uptake and rapid washout. The uptake in the liver was shown to be low, indicating that the probe is not primarily excreted *via* the hepatobiliary system. By contrast, the high measured probe concentration in kidney and urine indicated the rapid renal clearance of the agent. Overall, these properties represent a favourable pharmacokinetic profile of the elastin-specific molecular probe comparable to clinically used gadolinium-based contrast agents, such as gadobutrol.

### 4.2. Imaging Properties of MR Probes in a Clinical Setting

For the visualization of the vascular system in patients and the characterization of pathologies, different types of MR probes, including gadolinium-based and iron oxide-based agents have been investigated [2, 11]. In clinical practice, extravascular gadolinium-based contrast agents are the most commonly used imaging agents for applications, in which contrast enhancement is required. The most important clinical application of these imaging agents is the visualization of the different vascular phases, including the arterial, venous, and delayed phase with a high SNR and CNR. For the evaluation of patients, the most important clinical information for the characterization of pathologies are derived from these phases. If a novel agent probe is introduced into clinical practice, the probe should ideally enable imaging of these clinically important phases. The current clinically used MR contrast agents rapidly extravasate out of the arterial and venous lumen into the extracellular space following the early blood phase. In the context of cardiovascular imaging, current clinically used MR agents have been shown to accumulate in areas with fibrotic tissue, e.g., enabling myocardial scar imaging. However as this is a “passive” process not targeted against a specific protein or cell, they provide only indirect/unspecific and limited information about pathological processes when used for delayed enhancement.

In this study, we investigated the properties of a novel elastin-specific gadolinium-based molecular probe for the visualization of the clinically important early arterial phases. Importantly, all measurements were performed at the same clinically relevant dose (0.1 mmol/kg) with a single bolus administration. Additionally, all imaging was performed with clinically used MR sequences and at a clinical field strength of 3T. The elastin-specific gadolinium-based molecular probe demonstrated comparable imaging properties to the routinely used clinical contrast agent gadobutrol for the performance of an MR angiography. Following the intravenous injection, both agents were shown to demonstrate a comparable rapid renal clearance from the blood pool. Because of this property, the highest intravascular signal could be achieved during the first-pass following the intravenous administration. Additionally, both agents showed a similar SNR and CNR during the first-pass and a comparable washout pattern, which is in line with previous studies [5–8]. The SNR and CNR derived from the elastin-specific agent was slightly, however not significantly, higher compared to the enhancement derived from gadobutrol. This can be explained by the slightly higher relaxivity of the elastin-specific agent compared to gadobutrol [5, 9].

In summary, this study demonstrated that the elastin-specific molecular probe shows excellent signal properties for the acquisition of time-resolved first-pass and late 3D MR angiographies. Signal enhancement of the aorta was comparable to what was measured with the clinically used agent (gadobutrol) at the same dose. This indicates that first-pass angiography and tissue perfusion, e.g., for tumor evaluation could be performed with comparable SNR and CNR as with currently used clinical gadolinium-based agents. The main advantage of the elastin-specific probe, compared to unspecific clinically used probes, is that it can also be used for the visualization of specific pathological processes with the expression of (tropo-)elastin in delayed imaging.

### 4.3. Translation of Results into a Clinical Setting

This study has several advantages regarding the translation of the results into clinical applications: (1) All imaging were performed at a clinically relevant field strength, which allows direct translation to human applications. (2) The MR sequences used in this study are based on a clinical MR imaging protocol. (3) The molecular character and size of the used probe is directly comparable to clinically approved contrast agents, increasing the probability of a translation into clinical applications. Despite the prevalence of high-field scanners in previously published small animals MRI studies, clinical MRI systems are being increasingly used for preclinical research. This study was performed on a clinical 3T MR imaging system for several reasons. The effects on T1 and T2 relaxation, rotational correlation and signal properties of molecular probes, and potential sources of artefacts (fat, air/tissue interfaces) can vary substantially depending on the applied field strength (1.5–3T vs. >7T) and may therefore limit the direct translation of the findings to human application [12]. Additionally, when utilizing a clinical MRI system, protocols and imaging pulse sequences that are already highly optimized for clinical practice, can be adapted and scaled down for preclinical use. Subsequently, these sequences can easily be scaled back for clinical practice. The translation from preclinical to clinical studies is therefore greatly facilitated. 3T is chosen over 1.5T because of the increased SNR and CNR and thus higher spatial resolution achievable. A clinical single-loop microscopy coil was used to achieve the high signal levels needed for high-resolution small animal imaging.

### 4.4. Limitations

This study has several limitations. The MR probe was evaluated in an experimental small animal study. However, previous experimental studies have shown that this type of study can yield a good indication of how a MR agent could perform in a clinical setting. Secondly, a relatively small number of animals with defined doses of the MR agent was used. Additionally, a single acquisition protocol with a single regime of contrast injection was applied. This acquisition protocol was directly derived from the clinical imaging protocol and scaled down for this study. Imaging was performed only at 3T and therefore the results are not directly applicable to 1.5T scanners.

## 5. Conclusion

The novel elastin-specific molecular probe enables the performance of a contrast-enhanced first-pass and late 3D MR angiography with an intravascular contrast enhancement and image quality comparable to a clinically used extravascular contrast agent.

## Data Availability

The data used to support the findings of this study are available from the corresponding author upon request.

## Supplementary Materials

Supplementary Materials Supplementary Figure 1: (A) example of tubes with diluted gadobutrol of the approximated *in vivo* concentration ranging from 0.00125 mmol per ml to 0.00033 mmol per ml. We imaged with the same imaging set-up and MR sequences as in the *in vivo* experiments. (B) A close correlation of the *ex vivo* SNR and CNR measurements with the overall gadolinium concentration over a wide range of dilutions was found. Additionally, measurements in 3  mm tubes strongly correlated with measurements in 1 mm tubes. Supplementary Figure 2: the red regions indicate the region of interest (ROI) used for the assessment of the signal from the left ventricle. (A, B) The images show the first-pass MR angiography (TWIST) following the administration of the elastin agent and the clinically used agent gadobutrol. (A1, B1) Precontrast images at the level of the heart showed no relevant signal enhancement prior to the administration of the probes. (A2, B2) Immediately after the administration of the imaging agents, a high signal in the left and right ventricle can be appreciated during the first-pass. Visually, the signal intensity from both probes was comparable. (A3, B3) 30 seconds after the administration of the probes, a clear reduction of the signal at the level of the heart can be appreciated. (A4, B4) 60 seconds following the administration of the probes, a further decrease in signal can be appreciated. RV: right ventricle, LV: left ventricle.
