# Supplementary material for: Contrast-Enhanced Magnetic Resonance Angiography Using a Novel Elastin-Specific Molecular Probe in an Experimental Animal Model
Source: Contrast Media Mol Imaging. 2018 Oct 23;2018:9217456. doi: 10.1155/2018/9217456 (PMC6218789; doi:10.1155/2018/9217456)
Supplement: Supplementary Materials — Supplementary Figure 1: (A) example of tubes with diluted gadobutrol of the approximated in vivo concentration ranging from 0.00125 mmol per ml to 0.00033 mmol per ml. We imaged with the same imaging set-up and MR sequences as in the in vivo experiments. (B) A close correlation of the ex vivo SNR and CNR measurements with the overall gadolinium concentration over a wide range of dilutions was found. Additionally, measurements in 3 mm tubes strongly correlated with measurements in 1 mm tubes. Supplementary Figure 2: the red regions indicate the region of interest (ROI) used for the assessment of the signal from the left ventricle. (A, B) The images show the first-pass MR angiography (TWIST) following the administration of the elastin agent and the clinically used agent gadobutrol. (A1, B1) Precontrast images at the level of the heart showed no relevant signal enhancement prior to the administration of the probes. (A2, B2) Immediately after the administration of the imaging agents, a high signal in the left and right ventricle can be appreciated during the first-pass. Visually, the signal intensity from both probes was comparable. (A3, B3) 30 seconds after the administration of the probes, a clear reduction of the signal at the level of the heart can be appreciated. (A4, B4) 60 seconds following the administration of the probes, a further decrease in signal can be appreciated. RV: right ventricle, LV: left ventricle. [file 9217456.f1.docx]

Supplementary Material

Contrast-enhanced magnetic resonance angiography

using a novel elastin-specific molecular probe

in an experimental animal model

Phantom experiment

An additional phantom study was performed to test the sensitivity of the system setup. Gadobutrol was diluted to different in vivo concentration, ranging from 0.00125 mmol/ml to 0.00033 mmol/ml. The imaging set up and MR sequences were the same as in the in vivo experiments. A close correlation of ex vivo SNR and CNR measurements over a wide range of concentrations was measured. Additionally, measurements in 3 mm tubes strongly correlated with measurements in 1 mm tubes (Supplementary Figure 1).


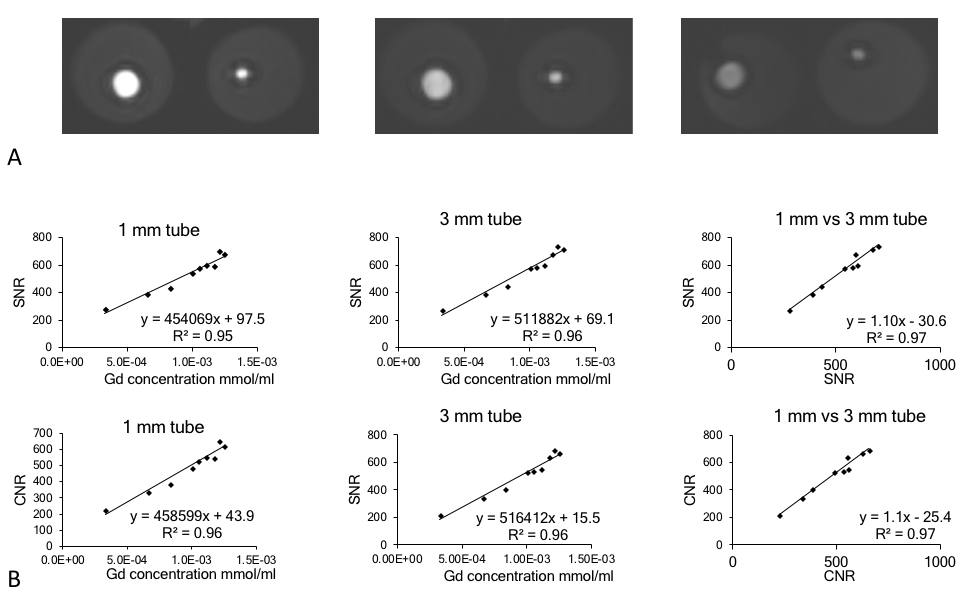


Supplementary Figure 1: A: Example of tubes with diluted gadobutrol of the approximated in vivo concentration ranging from 0.00125 mmol per ml to 0.00033 mmol per ml. We imaged with the same imaging set up and MR sequences as in the in vivo experiments. B: A close correlation of the ex vivo SNR and CNR measurements with the overall gadolinium concentration over a wide range of dilutions was found. Additionally, measurements in 3 mm tubes strongly correlated with measurements in 1 mm tubes.

To quantify the vascular signal enhancement, signal measurements were performed using a region of interest in the left ventricle of the heart as shown in Supplementary Figure 2.


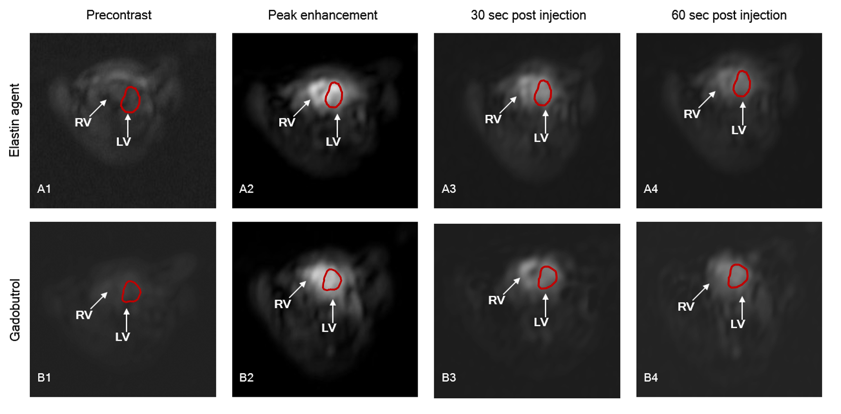


Supplementary Figure 2: The red regions indicate the region of interest (ROI) used for the assessment of the signal from the left ventricle. A, B: The images show the first-pass MR angiography (TWIST) following the administration of the elastin agent and the clinically used agent gadobutrol. A1, B1: Pre-contrast images at the level of the heart showed no relevant signal enhancement prior to the administration of the probes. A2, B2: Immediately after the administration of the imaging agents, a high signal in the left and right ventricle can be appreciated during the first-pass. Visually, the signal intensity from both probes were comparable. A3, B3: 30 seconds after the administration of the probes, a clear reduction of the signal at the level of the heart can be appreciated. A4, B4: 60 seconds following the administration of the probes a further decrease in signal can be appreciated. RV: right ventricle, LV: left ventricle.
